# Analysis of clinical factors affecting pregnancy outcomes after embryo transfer in patients with intrauterine adhesions

**DOI:** 10.3389/fmed.2025.1651805

**Published:** 2025-10-31

**Authors:** Jing Liu, Hui Chen, BeiBei Lin, Chen Wang, DeYing Ban, XiaoYing Zhong, HongXiang Sun

**Affiliations:** ^1^Reproductive and Genetic Hospital of CITIC-Xiangya, Changsha, China; ^2^Central South University Reproductive and Stem Cell Engineering Institute, Changsha, China

**Keywords:** intrauterine adhesions, embryo transfer, pregnancy outcome, clinical pregnancy rate, live birth rate

## Abstract

**Objective:**

This study aimed to investigate factors influencing embryo transfer success rates after hysteroscopic adhesiolysis in patients with intrauterine adhesions (IUAs).

**Methods:**

A retrospective analysis was conducted on the clinical data of 2,447 patients who underwent hysteroscopy and were diagnosed with intrauterine adhesions (IUAs) at our center from January 2023 to December 2023. All patients received adhesion separation surgery and underwent embryo transfer through assisted reproductive technology (ART) after the operation. The patients were divided into the non-pregnancy group (*n* = 955) and the pregnancy group (*n* = 1,492) based on pregnancy outcomes. The baseline characteristics, degree of intrauterine adhesions, type and duration of balloon placement, time interval from surgery to embryo transfer, number and type of transferred embryos, quality of transferred embryos, and endometrial thickness before transfer were compared between the two groups. Univariate and multivariate regression analysis methods were performed to identify factors affecting the success rate of embryo transfer.

**Results:**

Univariate analysis revealed significant associations between pregnancy outcomes and the followinf factors: age (OR = 0.91, *p* < 0.001), anti-Müllerian hormone (AMH, OR = 1.06, *p* < 0.001), infertility duration (OR = 0.96, *p* = 0.044), severe degree of intrauterine adhesion (OR = 0.47, *p* = 0.001), balloon placement time (OR = 1.01, *p* = 0.002), pre-transplant endometrial thickness (OR = 1.24, *p* < 0.001), frozen–thawed embryo transfer (OR = 0.38, *p* < 0.001), blastocyst transfer (OR = 1.92, *p* < 0.001), and transfer of high-quality embryos (OR = 1.30, *p* = 0.002) were significantly associated with pregnancy outcomes. Multivariate analysis further clarified the independent effects of age (OR = 0.92, *p* < 0.001), severe degree of intrauterine adhesion (OR = 0.31, *p* = 0.001), endometrial thickness before embryo transfer (OR = 1.19, *p* < 0.001), blastocyst transfer (OR = 2.03, *p* < 0.001), and transfer of high-quality embryos (OR = 1.36, *p* = 0.001) on pregnancy outcomes.

**Conclusion:**

Age, pre-transplant endometrial thickness, severe intrauterine adhesions, blastocyst transfer, and transfer of high-quality embryos are independent factors associated with pregnancy outcomes following intrauterine adhesion separation and subsequent embryo transfer.

## 1 Introduction

Intrauterine adhesions (IUAs) result from damage to the basal layer of the endometrium caused by various factors, leading to the formation of fibrous tissue and adhesion bands between the uterine walls. This condition alters the morphology of the uterine cavity, causing a range of clinical symptoms such as reduced menstrual flow, amenorrhea, infertility, and recurrent miscarriage. It significantly affects women’s reproductive physiology and mental health (1–3). Intrauterine adhesions are also a critical factor influencing pregnancy outcomes in women undergoing assisted reproductive technology (ART). Hysteroscopic adhesiolysis is the primary treatment for IUAs, aiming to restore the size and shape of the uterine cavity, promote endometrial repair, and reinstate normal menstruation and fertility (4, 5). Studies have reported that the live birth rate among patients with IUAs can reach 67.4% following hysteroscopic surgery. However, more severe adhesions are associated with poorer prognoses and often require multiple surgical interventions (6). With the recent adjustments to China’s fertility policy, an increasing number of women are seeking assisted reproductive technology (ART) to achieve pregnancy. However, a significant proportion of these women present with concomitant intrauterine adhesions, which may compromise the efficacy of ART and further complicate treatment outcomes. Improving the pregnancy success rate in this patient population has therefore become a critical clinical challenge requiring focused attention. Currently, there is limited research analyzing pregnancy outcomes following assisted reproductive technology (ART) in patients who have undergone surgery for intrauterine adhesions. This study aims to identify potential factors influencing embryo transfer outcomes after adhesion separation, thereby providing evidence to support clinical decision-making.

## 2 Objects and methods

### 2.1 Subjects

This study included 2,447 patients who underwent hysteroscopic adhesiolysis for intrauterine adhesions and subsequently completed embryo transfer at our center between January and December 2023, with a mean age of 34.03 ± 4.37 years. The study was approved by the institutional ethics committee under approval number LL-SC-2024-027.

Inclusion criteria: Patients eligible for this study met the following conditions: (a) diagnosis of intrauterine adhesions confirmed by hysteroscopy. (b) Patients who underwent embryo transfer at our center following surgical intervention.

Exclusion criteria: Presence of untreated unilateral or bilateral hydrosalpinx prior to embryo transfer. Concurrent untreated intrauterine pathology. Patients who underwent embryo transfer at external institutions. Individuals with incomplete clinical records.

**Figure 1:**
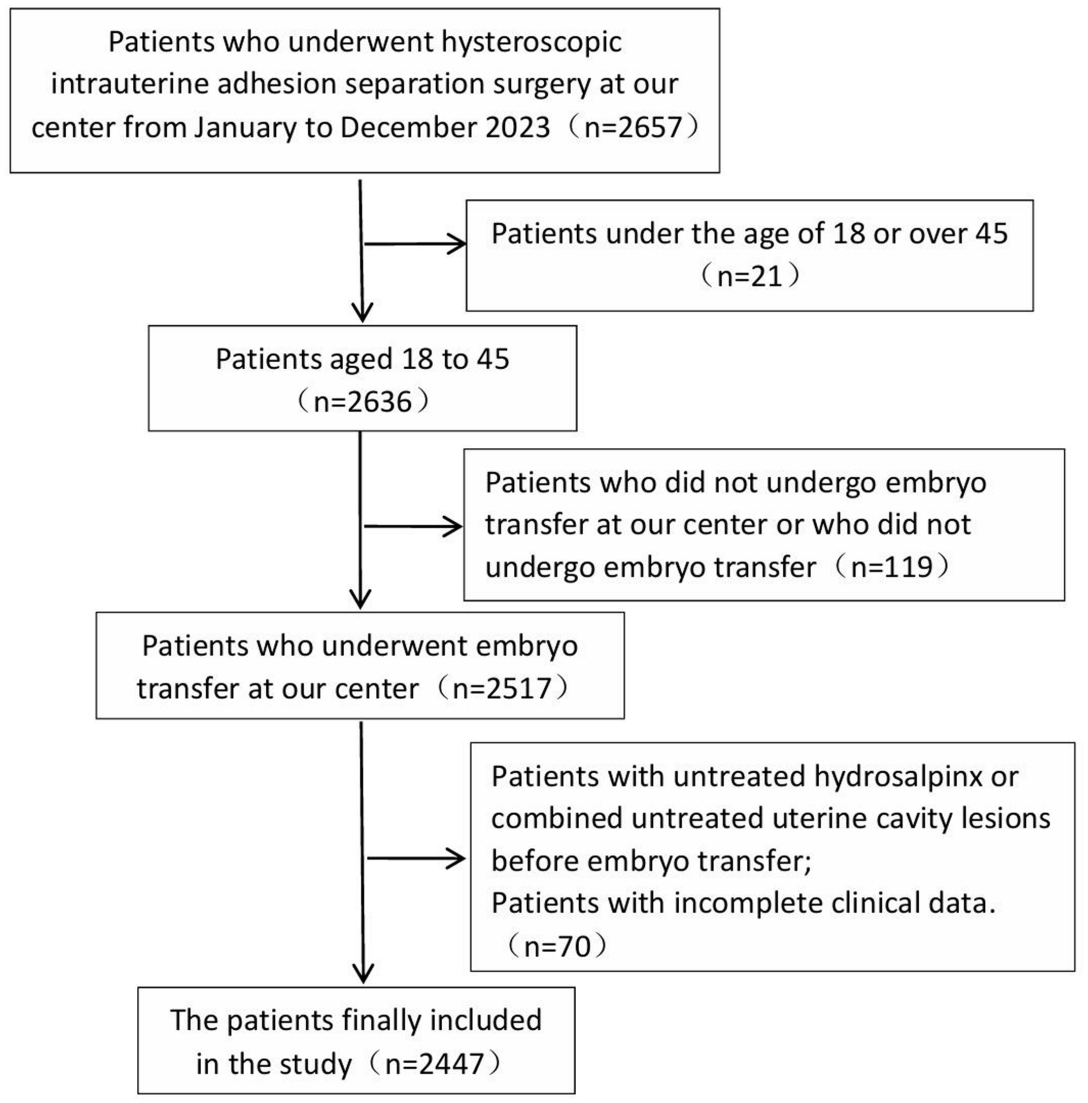
Inclusion of patients in the screening process.

Figure 1 illustrates the case screening process.

### 2.2 Methods

A retrospective case analysis was performed to collect and organize patient baseline characteristics, including age, body mass index (BMI), anti-Müllerian hormone (AMH) level, and duration of infertility, as well as the degree of intrauterine adhesion, type of balloon placement, balloon retention duration, interval between surgery and embryo transfer, number of embryos transferred, embryo type, whether high-quality embryos were transferred, and endometrial thickness prior to transfer. Based on the pregnancy outcome of the first embryo transfer cycle following intrauterine adhesiolysis, patients were classified into non-pregnancy and pregnancy groups.

Diagnosis and classification criteria for IUAs referred to the American fertility society (AFS) scoring and classification system (7): (1) Extent of IUAs: < 1/3 scored 1 point, 1/3–2/3 scored 2 points, > 2/3 scored 4 points; (2) type of adhesion: membranous adhesion scored 1 point, membranous and dense adhesion scored 2 points, dense adhesion scored 4 points; and (3) menstrual status: normal menstrual volume scored 0 points, decreased menstrual volume scored 2 points, and amenorrhea scored 4 points. The scores from the three categories were summed to calculate the total score, with 1–4 indicating mild adhesions, 5–8 indicating moderate adhesions, and 9–12 indicating severe adhesions.

#### 2.2.1 IUA surgery and postoperative treatment

Surgical Management and Postoperative Care of Intrauterine Adhesions The primary objective of intrauterine adhesion separation surgery is to excise fibrous adhesions and correct uterine cavity deformities caused by scar contracture. Currently, hysteroscopic adhesiolysis is the standard surgical approach for treating intrauterine adhesions (4, 5). The procedure is typically scheduled between 3 and 7 days after the completion of menstruation. Upon confirmation of intrauterine adhesions via hysteroscopy, adhesions are dissected using either cold scissors or a high-frequency electrosurgical device. This is accompanied by resection of fibrotic tissue to restore normal uterine morphology and cavity volume. In cases of moderate to severe adhesions, transabdominal ultrasound guidance may be used during surgery to enhance procedural safety and precision. Postoperatively, intrauterine administration of Gong’an Kang (a cross-linked sodium hyaluronate gel formulated for intrauterine use) is routinely performed to minimize the risk of re-adhesion. For moderate and severe cases, placement of an intrauterine balloon catheter is standard practice as an adjunctive measure to prevent recurrence. The selection of balloon type—such as pediatric urinary catheter balloons, disposable balloon uterine stents, or COOK balloons—is based on the extent of adhesions and the dimensions of the uterine cavity. Follow-up hysteroscopy is scheduled according to both the severity of adhesions and the type of balloon used: for patients with pediatric urinary catheter balloons, hysteroscopic removal is performed 7–14 days postoperatively, followed by a second evaluation 1 month later to assess endometrial recovery; for those with disposable balloon uterine stents or COOK balloons, hysteroscopy and balloon removal are conducted 1–3 months after surgery. Balloon placement may be omitted in patients with mild intrauterine adhesions. Additionally, postoperative hormonal therapy with estrogen and progestin is administered as clinically indicated, tailored to the severity of adhesions, to promote endometrial regeneration and functional restoration.

#### 2.2.2 Embryo quality scoring

The quality of cleavage-stage embryos was assessed according to the Peter cleavage-stage embryo scoring system. Embryos with more than seven cells and classified as Grade I or II were considered high-quality cleavage-stage embryos. Blastocyst quality was evaluated using the Gardner grading system, and blastocysts with a score of ≥4BB were defined as high-quality blastocysts.

#### 2.2.3 Pregnancy outcome indicators

The primary outcome was clinical pregnancy rate, defined as the number of clinical pregnancy cycles divided by the total number of embryo transfer cycles, multiplied by 100%. Clinical pregnancy was confirmed by transvaginal ultrasonography 26–28 days after embryo transfer through the visualization of an intrauterine gestational sac.

Secondary outcomes included live birth rate (number of live birth cycles/total transfer cycles × 100%), preterm birth rate (number of preterm birth cycles/total transfer cycles × 100%), miscarriage rate (number of miscarriage cycles/total transfer cycles × 100%), and ectopic pregnancy rate (number of ectopic pregnancy cycles/total transfer cycles × 100%). A live birth was defined as delivery following an intrauterine pregnancy of at least 28 weeks’ gestation resulting in a live-born neonate. Preterm birth was defined as delivery between 28 and 37 completed weeks of gestation with neonatal survival. All pregnancy losses occurring after the confirmation of clinical pregnancy—including spontaneous abortion, induced termination, and fetal demise—were classified as miscarriages.

### 2.3 Statistical methods

This study used SPSS 27.0 for statistical analysis. Normally distributed continuous variables were summarized using the mean ± standard deviation (SD), and group comparisons were performed using the two-sample independent t-test. Non-normally distributed continuous variables were presented as median (first quartile, third quartile) [M (Q₁, Q₃)], and differences between groups were assessed using the Mann–Whitney U-test. Categorical variables were expressed as frequency and percentage [*n* (%)], and comparisons were conducted using the chi-square test or Fisher’s exact test, as appropriate. A two-sided *p*-value of < 0.05 was considered indicative of statistical significance. Variables demonstrating significant differences in univariate analyses were subsequently included in univariate and multivariate logistic regression models to identify independent predictors.

## 3 Results

A total of 2,447 patients who underwent embryo transfer following hysteroscopic adhesiolysis were enrolled in this study. The overall clinical pregnancy rate was 61.0% (1,492/2,447), the live birth rate was 47.6% (1,164/2,447), the preterm birth rate was 6.5% (159/2,447), the ectopic pregnancy rate was 0.6% (14/2,447), and the miscarriage rate was 23.3% (569/2,447). Patients were stratified into three groups according to the severity of intrauterine adhesions: mild adhesion (*n* = 185, 7.56%), moderate adhesion (*n* = 2,142, 87.54%), and severe adhesion (*n* = 120, 4.90%). The clinical pregnancy rates were 60.5% (112/185), 62.1% (1,330/2,142), and 41.7% (50/120) in the respective groups, and the corresponding live birth rates were 44.3% (82/185), 49.0% (1,049/2,142), and 27.5% (33/120). Statistical analysis revealed statistically significant differences in both clinical pregnancy and live birth rates between the severe adhesion group and the combined mild and moderate adhesion groups (*p* < 0.05), as presented in Table 1.

**Table 1:** Comparison of clinical pregnancy rates and live birth rates among patients with varying degrees of intrauterine adhesions.

Intrauterine adhesions score	Case number	Number of clinical pregnancies	Clinical pregnancy rate	Number of live births	Live birth rate
Mild	185	112	60.5%^a	82	44.3%^a
Moderate	2,142	1,330	62.1%^a	1,049	49.0%^a
Severe	120	50	41.7%^b	33	27.5%^b
*χ* ^2^			19.9		21.81
*p*			<0.0001		<0.0001

Different superscript letters within the same column denote statistically significant differences between groups.

Clinical pregnancy rate comparison: Mild vs. Moderate: *χ*^2^ = 0.17, *p* = 0.683 (no statistically significant difference); Mild vs. Severe: *χ*^2^ = 9.28, *p* = 0.002 (statistically significant difference); Moderate vs. Severe: *χ*^2^ = 19.25, *p* < 0.0001 (statistically significant difference).

Live birth rate Comparison: Mild vs. Moderate: *χ*^2^ = 1.51, *p* = 0.219 (no statistically significant difference); Mild vs. Severe: *χ*^2^ = 8.73, *p* = 0.003 (statistically significant difference); Moderate vs. Severe: *χ*^2^ = 20.59, *p* < 0.0001 (statistically significant difference).

Based on the pregnancy outcomes of the first embryo transfer cycle following hysteroscopic adhesiolysis, patients were classified into non-pregnancy and pregnancy groups, comprising 955 and 1,492 cases, respectively. Table 2 presents an analysis of factors potentially influencing embryo transfer success. Statistically significant differences were observed between the two groups in the following variables: age, AMH levels, duration of infertility, degree of intrauterine adhesions, balloon retention duration, balloon type, interval from surgery to embryo transfer, embryo transfer cycle type (fresh vs. frozen–thawed embryos), stage of embryo development (cleavage-stage embryos vs. blastocysts), and whether high-quality embryos were transferred (*p* < 0.05). Specifically, patients in the pregnancy group were significantly younger than those in the non-pregnancy group (33.33 ± 4.11 years vs. 35.14 ± 4.53 years, *p* < 0.001), had greater endometrial thickness prior to transfer (10.10 mm vs. 9.60 mm, *p* < 0.001), and exhibited higher AMH levels (3.29 ng/mL vs. 2.67 ng/mL, *p* < 0.001). In contrast, no statistically significant differences were observed between the groups with respect to body mass index (BMI) or the number of embryos transferred.

**Table 2:** Status and differences between the non-pregnant group and the pregnant group.

Variables	Total (*n* = 2,447)	Non-pregnant group	Pregnant group	Statistic	*p*
		(*n* = 955)	(*n* = 1,492)		
Age, Mean ± SD	34.03 ± 4.37	35.14 ± 4.53	33.33 ± 4.11	T = 9.99	<0.001
BMI, Mean ± SD	22.28 ± 2.70	22.30 ± 2.64	22.27 ± 2.74	T = 0.23	0.819
AMH, M (Q₁, Q₃)	3.03 (1.72, 5.08)	2.67 (1.48, 4.60)	3.29 (1.96, 5.39)	Z = 6.22	<0.001
Duration of infertility, M (Q₁, Q₃)	2.00 (1.00, 3.00)	2.00 (1.00, 4.00)	2.00 (1.00, 3.00)	Z = 2.04	0.041
Intrauterine adhesions score, M (Q₁, Q₃)				Squared = 19.94	<0.001
Mild	185 (7.56)	73 (7.64)	112 (7.51)		
Moderate	2,142 (87.54)	812 (85.03)	1,330 (89.14)		
Severe	120 (4.90)	70 (7.33)	50 (3.35)		
Balloon placement time, M (Q₁, Q₃)	49.00 (38.00, 59.00)	47.00 (37.00, 57.00)	50.00 (39.00, 59.00)	Z = 3.33	<0.001
Balloon type, *n* (%)				Squared = 12.37	0.006
No	147 (6.01)	62 (6.49)	85 (5.70)		
Disposable balloon uterine stents	2036 (83.20)	766 (80.21)	1,270 (85.12)		
Pediatric catheter balloon	222 (9.07)	104 (10.89)	118 (7.91)		
COOK balloon	42 (1.72)	23 (2.41)	19 (1.27)		
Time to embryo transfer, M (Q₁, Q₃)	33.00 (14.00, 50.00)	35.00 (14.00, 52.00)	31.00 (14.00, 49.00)	Z = 2.39	0.017
Pre-transplant intimal thickness, M (Q₁, Q₃)	9.90 (9.00, 11.10)	9.60 (8.80, 10.70)	10.10 (9.10, 11.30)	Z = 7.24	<0.001
Number of embryos transferred, *n* (%)				Squared = 0.40	0.527
1 coin	1877 (76.71)	739 (77.38)	1,138 (76.27)		
2 pieces	570 (23.29)	216 (22.62)	354 (23.73)		
Transferred embryo cycles, *n* (%)				Squared = 11.77	<0.001
Fresh embryo	75 (3.06)	15 (1.57)	60 (4.02)		
Frozen and thawed embryos	2,372 (96.94)	940 (98.43)	1,432 (95.98)		
Embryo type, *n* (%)				Squared = 23.62	<0.001
Cleaved embryo	241 (9.85)	129 (13.51)	112 (7.51)		
Blastocyst	2,206 (90.15)	826 (86.49)	1,380 (92.49)		
Whether good quality embryos were transferred, *n* (%)				Squared = 9.32	0.002
No	848 (34.65)	366 (38.32)	482 (32.31)		
Yes	1,599 (65.35)	589 (61.68)	1,010 (67.69)		

t: t-test, Z: Mann–Whitney test, *χ*^2^: Chi-square test. SD: standard deviation, M: Median, Q₁: 1st Quartile, Q₃: 3st Quartile.

Based on the findings in Table 2, several potential factors associated with pregnancy outcomes were identified. Univariate and multivariate logistic regression analyses were subsequently performed to investigate the risk factors influencing pregnancy outcomes. As presented in Table 3, univariate analysis revealed that age (OR = 0.91, *p* < 0.001), AMH level (OR = 1.06, *p* < 0.001), duration of infertility (OR = 0.96, *p* = 0.044), severe intrauterine adhesions (OR = 0.47, *p* = 0.001), balloon retention time (OR = 1.01, *p* = 0.002), endometrial thickness prior to embryo transfer (OR = 1.24, *p* < 0.001), frozen–thawed embryo transfer (OR = 0.38, *p* < 0.001), blastocyst transfer (OR = 1.92, *p* < 0.001), and transfer of high-quality embryos (OR = 1.30, *p* = 0.002) were all significantly associated with pregnancy outcomes. Following adjustment for potential confounding variables, multivariate logistic regression analysis confirmed that age (OR = 0.92, *p* < 0.001), severe intrauterine adhesions (OR = 0.31, *p* = 0.001), endometrial thickness prior to embryo transfer (OR = 1.19, *p* < 0.001), blastocyst transfer (OR = 2.03, *p* < 0.001), and transfer of high-quality embryos (OR = 1.36, *p* = 0.001) were independent predictors of pregnancy outcomes. Specifically, embryo transfer success rates were significantly lower among older patients and those with severe intrauterine adhesions, whereas higher success rates were observed in patients with greater pre-transfer endometrial thickness, those undergoing blastocyst transfer, and those receiving high-quality embryos. These findings provide valuable insights for the clinical assessment of pregnancy success following embryo transfer.

**Table 3:** Analysis of risk factors for pregnancy outcomes (logistic regression analysis).

Variables	Single factor					Multiple factors				
	Beta.	S. E	Z	*p*	OR (95%CI)	Beta.	S. E	Z	*p*	OR (95%CI)
Age (year)	−0.10	0.01	−9.79	<0.001	0.91 (0.89–0.92)	−0.08	0.01	−7.69	<0.001	0.92 (0.90–0.94)
AMH (ng/ml)	0.06	0.01	4.51	<0.001	1.06 (1.03–1.09)	0.02	0.01	1.39	0.165	1.02 (0.99–1.04)
Duration of infertility (year)	−0.04	0.02	−2.02	0.044	0.96 (0.93–0.99)	−0.01	0.02	−0.70	0.487	0.99 (0.95–1.03)
Intrauterine adhesions score										
Mild					1.00 (Reference)					1.00 (Reference)
Moderate	0.07	0.16	0.42	0.677	1.07 (0.78 to 1.45)	−0.55	0.31	−1.74	0.081	0.58 (0.31–1.07)
Severe	−0.76	0.24	−3.20	0.001	0.47 (0.29–0.74)	−1.18	0.37	−3.21	0.001	0.31 (0.15–0.63)
Balloon placement time (d)	0.01	0.00	3.07	0.002	1.01 (1.01 to 1.01)	0.00	0.00	0.71	0.479	1.00 (1.00–1.01)
Type of balloon										
No					1.00 (Reference)					1.00 (Reference)
Disposable balloon uterine stents	0.19	0.17	1.10	0.272	1.21 (0.86–1.70)	0.41	0.21	1.94	0.052	1.51 (1.00–2.28)
Pediatric catheter balloon	−0.19	0.21	−0.88	0.378	0.83 (0.54–1.26)	0.18	0.26	0.68	0.494	1.20 (0.71–2.01)
COOK balloon	−0.51	0.35	−1.44	0.150	0.60 (0.30–1.20)	−0.37	0.38	−0.96	0.336	0.69 (0.33–1.46)
Postoperative time to embryo transfer (d)	−0.00	0.00	−1.71	0.087	1.00 (1.00 to 1.00)	0.00	0.00	0.18	0.855	1.00 (1.00–1.00)
Endometrial thickness (mm) before transplantation	0.21	0.03	7.43	<0.001	1.24 (1.17–1.31)	0.17	0.03	5.56	<0.001	1.19 (1.12–1.26)
Transfer embryo cycle										
Fresh embryos					1.00 (Reference)					1.00 (Reference)
Frozen–thawed embryos	−0.97	0.29	−3.31	<0.001	0.38 (0.22–0.67)	−0.26	1.01	−0.25	0.799	0.77 (0.11–5.56)
Embryo type										
Cleavage embryo					1.00 (Reference)					1.00 (Reference)
Blastocyst	0.65	0.14	4.80	<0.001	1.92 (1.47–2.51)	0.71	0.15	4.72	<0.001	2.03 (1.51–2.73)
Whether to transfer good-quality embryos										
No					1.00 (Reference)					1.00 (Reference)
Yes	0.26	0.09	3.05	0.002	1.30 (1.10 ~ 1.54)	0.31	0.09	3.29	0.001	1.36 (1.13–1.63)

## 4 Discussion

This study demonstrated that age is an independent factor influencing pregnancy outcomes (OR = 0.92, 95% CI = 0.90–0.94, *p* < 0.001). As patient age increases, the likelihood of a successful pregnancy decreases. Therefore, for infertile patients undergoing intrauterine adhesion surgery, early initiation of assisted reproductive technology following surgery may improve pregnancy outcomes.

Multiple studies have demonstrated that the severity of intrauterine adhesions significantly influences pregnancy outcomes, with more severe adhesions associated with lower pregnancy success rates (6). The findings of this study further confirm that patients with severe intrauterine adhesions exhibit a significantly reduced pregnancy rate (OR = 0.31, 95% CI = 0.15–0.63, *p* = 0.001). In addition, the more severe the degree of intrauterine adhesions, the higher the postoperative recurrence rate. Preventing the recurrence of adhesions after intrauterine adhesion separation is a difficult and key issue in both domestic and international research, especially in patients with severe intrauterine adhesions. Currently, the main preventive measures include placing balloon stents, intrauterine devices, biological glue-like materials in the uterine cavity, and postoperative oral administration of estrogen and progesterone therapy (5). The gynecological minimally invasive surgery team at this center primarily uses a combination of intrauterine injection of cross-linked sodium hyaluronate gel and placement of intrauterine balloons to prevent recurrence of intrauterine adhesions. The surgical team individualizes balloon selection based on each patient’s uterine cavity volume and extent of adhesions. This approach offers the key advantage of enabling precise matching of balloon size and type, thereby providing optimal mechanical support within the uterine cavity while minimizing endometrial compression caused by improperly sized balloons, which could otherwise impair endometrial regeneration and restoration of normal uterine morphology. For pediatric urinary catheter balloons, balloon volume can be adjusted by varying the volume of injected fluid, allowing for tailored expansion. These balloons are typically inserted under B-ultrasound guidance and retained for an average of 1 to 2 weeks postoperatively. Hysteroscopic evaluation is performed approximately 1 month after balloon removal to assess endometrial recovery. In contrast, the optimal duration of intrauterine balloon stent retention remains undefined and lacks standardized guidelines. Research shows (8) that the repair of the endometrium after hysteroscopic adhesiolysis usually takes 1 to 3 months. If the support time of the barrier after the operation is too short or insufficient, new adhesion bands may form before the endometrium is fully repaired. The mechanism of action of the intrauterine balloon stent is to expand the uterine cavity and form a physical barrier between the uterine wound surfaces. Its triangular structure matches the anatomical shape of the uterine cavity, which can achieve better adhesion when separating the uterine inner wall, increase the contact area, and thus more effectively restore the normal shape of the uterine cavity and reduce the risk of adhesion. In addition, the stent is made of silicone, which has good biocompatibility and high safety and can, to some extent, reduce the occurrence of related complications. Cumulative evidence from multiple clinical studies (9–11) demonstrates that extending the duration of balloon stent placement after hysteroscopic adhesiolysis is significantly associated with a lower risk of intrauterine adhesion recurrence, without a corresponding increase in the incidence of intrauterine bacterial infection. This study utilized two types of intrauterine devices: disposable balloon uterine stents and COOK balloons. The median duration of balloon stent placement was 49 days (range: 38–59 days), and statistical analysis revealed no significant association with adverse effects on pregnancy outcomes (*p* = 0.479). Furthermore, neither the type of balloon used nor the time interval between surgery and embryo transfer demonstrated a significant impact on pregnancy outcomes (*p* > 0.05; *p* = 0.855).

The findings of this study demonstrate that endometrial thickness prior to embryo transfer was significantly greater in the pregnancy group compared to the non-pregnancy group (*p* < 0.001). Multivariate regression analysis revealed that pre-transfer endometrial thickness is an independent predictor of pregnancy outcome (OR = 1.19, 95% CI: 1.12–1.26, *p* < 0.001), with a positive correlation observed between endometrial thickness and pregnancy rate—indicating that higher endometrial thickness is associated with an increased likelihood of pregnancy. A large-scale study from the United Kingdom involving 25,767 fresh embryo transfer cycles (12) demonstrated that endometrial thickness is significantly associated with both live birth rate and miscarriage rate. Using statistical modeling, the optimal threshold for endometrial thickness was identified as 10 mm, and this association remained robust after adjustment for potential confounding factors, including maternal age, number of retrieved oocytes, number of embryos transferred, and embryo transfer strategy. A Canadian study (13) encompassing 43,383 fresh embryo transfer cycles and 53,377 frozen–thawed embryo transfer cycles demonstrated that in fresh cycles, the live birth rate increased significantly with greater endometrial thickness up to the 10–12 mm range. In contrast, for frozen–thawed embryo transfer cycles, the live birth rate plateaued once the endometrial thickness reached 7–10 mm. However, an endometrial thickness below 6 mm was consistently associated with a marked reduction in live birth rates in both fresh and frozen–thawed embryo transfer cycles. A large-scale study from the United States (14) analyzed data from 244,001 frozen–thawed embryo transfer cycles (including 100,419 cycles with preimplantation genetic testing [PGT] and 96,249 without PGT) and 47,333 fresh embryo transfer cycles. The analysis demonstrated that across all cycle types, endometrial thickness was positively associated with live birth rate up to a threshold of 9 mm. Beyond this point, further increases in endometrial thickness yielded minimal gains in live birth rate. Prior to reaching 9 mm, each additional 1 mm increase in endometrial thickness was associated with a 19% relative increase in live birth rate in the PGT-utilizing frozen–thawed group (OR = 1.19, 95% CI: 1.06–1.22), a 13% increase in the non-PGT frozen–thawed group (OR = 1.13, 95% CI: 1.09–1.16), and a 15% increase in the fresh embryo transfer group (OR = 1.15, 95% CI: 1.09–1.20). Therefore, for patients following surgical treatment of intrauterine adhesions, excessive emphasis on endometrial thickness is not warranted. Once the endometrium reaches an adequate threshold, embryo transfer should be performed at the earliest feasible opportunity to shorten the treatment cycle and enhance the efficiency of assisted reproductive technology.

Embryo transfer strategies following hysteroscopic adhesiolysis represent a common clinical dilemma in assisted reproductive technology (ART). The choice between fresh and frozen–thawed embryo transfer has long been a subject of debate. Accumulating evidence (15–17) indicates that, compared with fresh embryo transfer, frozen–thawed embryo transfer is associated with higher implantation rates, clinical pregnancy rates, and live birth rates, as well as more favorable obstetric and perinatal outcomes, including lower risks of ectopic pregnancy and ovarian hyperstimulation syndrome (OHSS). However, it may be linked to an increased risk of gestational hypertension and macrosomia. The results of the univariate analysis in this study indicated that frozen–thawed embryo transfer was a risk factor affecting pregnancy outcomes (OR = 0.38, 95% CI: 0.22–0.67, *p* < 0.001), which was inconsistent with the conclusions of the above-mentioned studies. This discrepancy may stem from data bias, particularly due to the marked imbalance in sample sizes—only 75 patients were included in the fresh embryo transfer group, compared to 2,372 in the frozen–thawed group. Furthermore, multivariate logistic regression analysis, after adjusting for potential confounding factors, showed no statistically significant difference in pregnancy outcomes between the two transfer methods. Therefore, these findings warrant validation through future studies with larger, more balanced sample sizes.

Tinn Teh W et al. (18) demonstrated that the fresh embryo transfer group had significantly higher live birth rates (19.13% vs. 14.13%) and clinical pregnancy rates (22.48% vs. 16.25%) compared to the frozen–thawed embryo transfer group (*p* < 0.001). Multivariate analysis adjusting for potential confounding factors further confirmed that women undergoing frozen–thawed embryo transfer had a significantly lower likelihood of live birth relative to those receiving fresh embryo transfer (OR = 0.76, 95% CI: 0.68–0.86, *p* < 0.001). Another multicenter randomized controlled trial conducted in the UK (19) enrolled 616 patients, who were randomly assigned to either the fresh embryo transfer group (*n* = 309) or the frozen embryo transfer group (*n* = 307). The results showed no statistically significant differences between the two groups in terms of live birth rate, clinical pregnancy rate, miscarriage rate, or incidence of ovarian hyperstimulation syndrome (OHSS); however, the frozen embryo transfer group incurred higher treatment costs. A recent multicenter (20), randomized controlled trial demonstrated that among women with low *in vitro* fertilization success potential—defined as the retrieval of ≤9 oocytes or poor ovarian reserve (antral follicle count <5 or AMH < 8.6 mmol/L)—the live birth rate was significantly higher in the fresh embryo transfer group compared to the frozen–thawed embryo transfer group. Therefore, there is currently no definitive consensus regarding the choice between fresh and frozen–thawed embryo transfer. Clinical decisions should be individualized according to patient-specific characteristics to maximize the likelihood of successful embryo implantation and pregnancy outcomes. Therefore, there is currently no definitive consensus regarding the choice between fresh and frozen–thawed embryo transfer. Individualized strategies should be developed according to patient-specific characteristics to maximize the likelihood of successful implantation and clinical pregnancy outcomes (21, 22).

This study demonstrates that blastocyst transfer is associated with a significantly higher pregnancy success rate compared to cleavage-stage embryo transfer (OR = 2.03, 95% CI: 1.51–2.73, *p* < 0.001), a finding consistent with the systematic review and meta-analysis by Glujovsky D et al. (23). Their analysis, which included 32 randomized controlled trials involving 5,821 couples, reported that in fresh cycle embryo transfer cycles, both live birth rates and clinical pregnancy rates were significantly higher in the blastocyst transfer group than in the cleavage-stage embryo transfer group. A study by Holden EC et al. (24) included women who underwent frozen–thawed embryo transfer (FET) between 2004 and 2013. The cohort comprised 118,572 women who received blastocyst-stage FET and 117,619 who received cleavage-stage FET. After adjusting for potential confounding factors, the study found that, compared with cleavage-stage FET, blastocyst FET was associated with a 49% higher live birth rate (OR = 1.49, 95% CI: 1.44–1.54). Furthermore, blastocyst FET was associated with a 68% increase in clinical pregnancy rate (OR = 1.68, 95% CI: 1.63–1.74) and a 16% increase in preterm birth rate (OR = 1.16, 95% CI: 1.06–1.27), with no significant difference in birth weight observed. This study demonstrates that the transfer of high-quality embryos is an independent predictor of improved pregnancy outcomes (OR = 1.36, 95% CI: 1.13–1.63, *p* = 0.001), a finding consistent with previously published studies (25, 26). High-quality embryo transfer is associated with higher clinical pregnancy and live birth rates, whereas poor-quality embryos are linked to an increased risk of placenta previa during pregnancy.

## 5 Strengths and limitations

This study represents the largest analysis to date in terms of sample size, examining pregnancy outcomes following hysteroscopic adhesiolysis and subsequent embryo transfer. As our institution is a specialized reproductive center, follow-up of pregnancy outcomes primarily relies on telephone interviews, resulting in incomplete ascertainment of data on maternal pregnancy complications and neonatal morbidities. Consequently, a comprehensive analysis of live birth rates was not performed. Due to the retrospective design, the study may be subject to confounding factors and selection bias. Notably, among the included patients, the number of those undergoing fresh embryo transfer was substantially lower than that of those receiving frozen–thawed embryo transfer. This imbalance is largely attributable to current clinical practice at our center: for patients with moderate to severe intrauterine adhesions, the majority are managed with a freeze-all strategy, allowing oocyte retrieval and embryo cryopreservation to occur during the endometrial recovery period, thereby optimizing endometrial receptivity and reducing the overall treatment duration. Future research should focus on well-designed prospective studies with larger sample sizes and high-quality data collection to further elucidate the optimal assisted reproductive strategy following hysteroscopic adhesiolysis.

## 6 Conclusion

In conclusion, for patients undergoing hysteroscopic adhesiolysis followed by assisted reproductive technology (ART) treatment, age, endometrial thickness, blastocyst-stage transfer, and high-quality embryo transfer are independent predictors of embryo transfer pregnancy outcomes. Therefore, once the postoperative endometrium reaches adequate conditions for transfer, early transfer of high-quality blastocysts should be prioritized to improve clinical pregnancy rates. These findings may serve as a valuable reference for developing individualized embryo transfer strategies following hysteroscopic adhesiolysis.

## Data Availability

The original contributions presented in the study are included in the article/supplementary material, further inquiries can be directed to the corresponding author.
